# Defining patient outcomes in stage IV colorectal cancer: a prospective study with baseline stratification according to disease resectability status

**DOI:** 10.1038/sj.bjc.6605508

**Published:** 2010-01-19

**Authors:** D J Watkins, I Chau, D Cunningham, S S Mudan, N Karanjia, G Brown, S Ashley, A R Norman, A Gillbanks

**Affiliations:** 1 Department of Medicine, Royal Marsden Hospital, London & Surrey, UK; 2 Department of Surgery, Royal Marsden Hospital, London & Surrey, UK; 3 Department of Surgery, The Royal Surrey County Hospital, Guildford, UK; 4 Department of Diagnostic Imaging, Royal Marsden Hospital, London & Surrey, UK

**Keywords:** Stage IV colorectal cancer, liver metastases, peri-operative chemotherapy, metastectomy, liver resection

## Abstract

**Background:**

Stage IV colorectal cancer encompasses a broad patient population in which both curative and palliative management strategies may be used. In a phase II study primarily designed to assess the efficacy of capecitabine and oxaliplatin, we were able to prospectively examine the outcomes of patients with stage IV colorectal cancer according to the baseline resectability status.

**Methods:**

At enrolment, patients were stratified into three subgroups according to the resectability of liver disease and treatment intent: palliative chemotherapy (subgroup A), conversion therapy (subgroup B) or neoadjuvant therapy (subgroup C). All patients received chemotherapy with capecitabine 2000 mg m^–2^ on days 1–14 and oxaliplatin 130 mg m^–2^ on day 1 repeated every 3 weeks. Imaging was repeated every four cycles where feasible liver resection was undertaken after four or eight cycles of chemotherapy.

**Results:**

Of 128 enrolled patients, 74, 22 and 32 were stratified into subgroups A, B and C, respectively. Attempt at curative liver resection was undertaken in 10 (45%) patients in subgroup B and 19 (59%) in subgroup C. The median overall survival was 14.6, 24.5 and 52.9 months in subgroups A, B and C, respectively. For patients in subgroups B and C who underwent an attempt at curative resection, 3-year progression-free survival was 10% in subgroup B and 37% for subgroup C.

**Conclusions:**

This prospective study shows the wide variation in outcome according to baseline resectability status and highlights the potential clinical value of a modified staging system to distinguish between these patient subgroups.

The overall survival of patients with advanced colorectal cancer has improved from a median of approximately 14 months achieved with 5-FU/LV alone (Douillard *et al*, 2000; de Gramont *et al*, 2000) to more than 19 months with the use of combination and sequential cytotoxic therapies (Goldberg *et al*, 2004; Tournigand *et al*, 2004; Souglakos *et al*, 2006). More recently, targeted therapeutics have shown further incremental gains (Hurwitz *et al*, 2004; Jonker *et al*, 2007; Van Cutsem *et al*, 2007) with the prospect of extending survival beyond 24 months in the advanced disease setting (Grothey *et al*, 2007). For the majority, treatment remains of palliative benefit, with the possibility of cure restricted only to those patients with disease suitable for surgical resection.

Before the advent of combination chemotherapy, the role of metastectomy was limited to those patients who initially presented with disease amenable to surgical resection. However, the high tumour response rates achieved with modern chemotherapeutics now enable a further proportion of patients with initially inoperable disease to be converted to an operable status and undergo liver resection with curative intent. Reports published by Bismuth *et al* (1996) and Adam *et al* (2004a) have shown the potential long-term survival achievable through the use of down-sizing chemotherapy or ‘conversion therapy’ (Khatri *et al*, 2007) and liver resection, with 5-year survival rates of 33% (Adam *et al*, 2004a).

Published data suggest that the ability to undertake liver resection is a significant determinant of patient outcome in stage IV colorectal cancer. However, this has never been formally examined in a prospective study.

At the time of planning this study, one of the treatment options for patients with inoperable metastatic disease isolated to the liver was infused 5-fluorouracil, leucovorin and oxaliplatin (FOLFOX)(NICE, 2002). The combination of capecitabine and oxaliplatin (CapOx) represented a convenient alternative regimen, with phase II data available to support its use in the advanced disease setting (Borner *et al*, 2002). During the course of the study, phase III data showing the non-inferiority of CapOx compared with FOLFOX-4 have become available (Cassidy *et al*, 2008; Rothenberg *et al*, 2008). The objectives of this study were to further assess the safety and efficacy of capecitabine and oxaliplatin in the palliative disease setting and also in the peri-operative setting for patients with potentially resectable liver disease. The study design enabled the prospective evaluation of treatment outcomes according to the baseline disease resectability status and provides a unique data set in this patient population.

## Methods

This single-arm phase II study recruited patients referred to the Gastrointestinal Cancer Unit at the Royal Marsden Hospital, London and Sutton. The study was approved by the local Ethics Committee and written informed consent was obtained from all patients.

### Patients

Eligible patients diagnosed with advanced colorectal cancer were aged 18 years or older and had not received chemotherapy for advanced disease. Other requirements included unidimensional measurable disease, WHO (World Health Organisation) performance status 0–2, adequate bone marrow function, adequate liver function (bilirubin <1.5 × ULN), calculated creatinine clearance >50 mls min^–1^ and life expectancy of >12 weeks. Patients were excluded if they had clinically significant active cardiac disease (congestive cardiac failure, coronary artery disease, cardiac arrhythmia) or myocardial infarction within the last 12 months. Previous adjuvant therapy was allowed, including the administration of oxaliplatin-containing regimens. Patients with significant symptoms of peripheral neuropathy were excluded. Patients with either resectable or non-resectable metastatic disease sites were eligible for study enrolment.

### Study design and treatment

**Figure 1:**
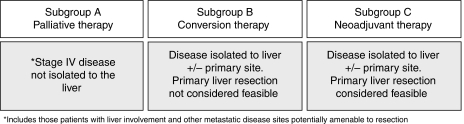
Prospective classification according to baseline resectability status. At the time of study entry, enrolled patients were classified into one of three subgroups on the basis of the feasibility of undertaking primary liver resection.

For the purpose of outcome analysis, patients were stratified into one of three subgroups according to their disease resectability status. This was undertaken prospectively by the investigator at the time of study enrolment as outlined in Figure 1. Stratification was based on the considered feasibility of proceeding directly to primary liver resection based on radiological findings, clinical details and multi-disciplinary meeting (MDM) discussion. The aim of stratification was to identify patient subgroups receiving palliative chemotherapy (subgroup A), conversion therapy (subgroup B) or neoadjuvant therapy (subgroup C). Stratification was solely undertaken to allow the outcomes of each patient subgroup to be evaluated independently and had no influence on the care that patients received while participating in the study.

Pre-treatment CT imaging was undertaken within 28 days before commencing study treatment. In addition, patients with liver-only metastases underwent contrast-enhanced MRI imaging of the liver and were discussed in a specialist hepatic MDM. Patients received CapOx chemotherapy consisting of oxaliplatin 130 mg m^–2^ on day 1 and capecitabine 1000 mg m^–2^ bd on days 1–14 repeated every 21 days. Patients aged 75 years or over received a reduced starting dose (oxaliplatin 100 mg m^–2^ and capecitabine 1500 mg m^–2^). Dose reductions of oxaliplatin were instituted for grade ⩾3 haematological toxicity (on day of treatment) and grade 3 peripheral neuropathy or grade 2 neuropathy persisting between cycles. Capecitabine dose reductions were instituted for non-haematological capecitabine toxicities of ⩾grade 2. Treatment was continued for up to a total of eight cycles. In responding patients, further cycles could be delivered at the discretion of the investigator. CT (and MRI if liver-only metastases) response assessment was undertaken after every four cycles. Patients considered to have potentially resectable disease were re-discussed in the hepatic MDM after every four cycles of treatment. The decision to use surgery or RFA was based on the MDM discussion and was not protocol specified. Liver resection was considered in cases in which macroscopic clearance of disease with clear margins was thought possible, while maintaining adequate residual liver tissue. Surgical resections were planned on the basis of both CT and MRI findings. The preferred timing for liver resection was after four cycles of CapOx chemotherapy, with an interval of 3–6 weeks between the end of chemotherapy and surgery recommended. In cases in which disease remained inoperable, a further four cycles of CapOx could be delivered before re-evaluation. Individual liver lesions that showed a complete response to chemotherapy on liver MRI (and intra-operative ultrasound where undertaken) were not resected. In cases in which resection of both the primary tumour and liver disease was necessary, this could be undertaken as either a combined procedure or sequentially with four cycles of CapOx delivered between each procedure. The use of long-course pre-operative pelvic chemoradiotherapy was permitted in patients with locally advanced rectal tumours. When used, this was administered after an initial four cycles of CapOx chemotherapy. Patients undergoing liver resection after four cycles of CapOx received a further four cycles of post-operative chemotherapy. After the completion of study therapy, patients were followed up on at least a 3-monthly basis.

### Statistical analysis

The primary outcome measure was radiological response rate after four cycles of CapOx chemotherapy. A response rate of 50% was considered acceptable and a response rate of 35% unacceptable. Using a minimax design (Simon, 1989) and a one-sided α of 0.05, a sample size of 117 allowed the exclusion of a radiological response rate of less than 35% with 95% power. Planned recruitment was 130 patients to allow for 10% of patients being non-assessable. Secondary outcome measures included progression-free survival (PFS), overall survival (OS), proportion of patients undergoing liver resection, chemotherapy-related toxicity and 60-day all-cause mortality.

For all patients, progression-free survival was calculated from the date of trial entry until disease progression, post-operative recurrence or death from any cause. Overall survival was calculated from the date of trial entry until death from any cause or censored at last follow-up. Both PFS and OS were estimated using the Kaplan–Meier method (Kaplan and Meier, 1958). The objective response rate was assessed by CT according to RECIST criteria (Therasse *et al*, 2000). Owing to a significant proportion of patients undergoing liver resection after four cycles of CapOx, radiological responses were not confirmed by repeat imaging. Toxicities were evaluated and recorded using the National Cancer Institute Common Toxicity Criteria version 2. A *post hoc* univariate and step-up multivariate Cox regression analysis was undertaken to examine for prognostic variables in all enrolled patients and in the liver-only subgroups (B and C). Factors included in the analysis for all patients were disease site (primary, local, liver, peritoneal, nodal, bone, lung), number of metastatic sites (1 *vs* >1), subgroup (A *vs* B *vs* C), age (<60 *vs* >60), PS (2 *vs* 0.1), disease-free interval (<12 *vs* >12 months), synchronous presentation (Y *vs* N), CEA (<200 *vs* >200), alk phos (<300 *vs* >300), LDH (<ULN *vs* >ULN), WCC (<10 *vs* >10), Hb (>11 *vs* <11) and platelets (<400 *vs* >400). For the analysis of liver-only patients, largest metastasis (<5 *vs* >5 cm) and number of liver metastases (1 *vs* >1) were also included. PS and Alk phos were excluded in the liver-only group because of low numbers. The data set was locked and analysed in October 2009 with a median follow-up of 60 months.

## Results

### Patient characteristics

Between September 2002 and April 2006, a total of 128 patients were recruited, with 74, 22 and 32 patients allocated to subgroups A, B and C, respectively.

The primary reasons for patients to be considered for conversion therapy (subgroup B *n*=22) were large metastasis (>5 cm) in five patients, multiple metastases (>4) in 12 cases, locally advanced primary in three patients and ill-placed lesions in two patients.

The patient characteristics for each subgroup are shown in Table 1. Patients in subgroup C were less likely to have presented with synchronous metastatic disease and had a longer interval between primary diagnosis and presentation with metastatic disease. The median number of metastatic liver deposits was lower in subgroup C compared with subgroup B, with a median of 2 (range 1–5) and 4 (range 1–15), respectively.

### Toxicities

The commonest grade III/IV chemotherapy-related toxicities were diarrhoea, neutropaenia and palmar plantar syndrome (Supplementary Table 2). Oxaliplatin was discontinued early in four patients because of peripheral neuropathy. Four patients discontinued capecitabine because of cardiac chest pain; raltitrexed was substituted for capecitabine in three of these cases. Two patients suffered fatal pulmonary thrombo-embolic events while on treatment. One patient suffered a fatal myocardial infarction and another patient suffered a non-fatal myocardial infarction. One death occurring during the first cycle of therapy was attributable to diarrhoea and dehydration. No deaths were attributable to neutropaenic sepsis, and 60-day all-cause mortality was 3.1%.

### Radiological response and resection rate

The median number of cycles of chemotherapy delivered was eight. Six patients (4.7%) were non-evaluable for response. By intention to treat, the overall radiological response rate as assessed by CT was 52.3% (95% CI: 43–61%); complete response 8.6%, partial response 43.8%, stable disease 30.5% and progressive disease 12.5%. The radiological response rate and liver resection rate for each subgroup are summarised in Table 2. As would be expected, a higher proportion of patients in subgroup C underwent an attempt at liver resection than those in subgroup B. Two patients in subgroup A underwent resection of pulmonary metastases and another patient had pulmonary and liver metastases resected. For patients undergoing an attempt at liver resection, the median interval between the end of pre-operative chemotherapy and surgery was 7.6 weeks (range 4–24 weeks).

### Liver resections

**Figure 2:**
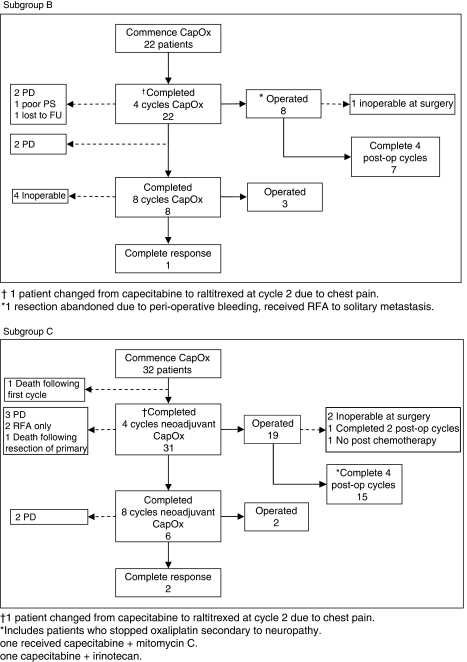
Flow diagram indicating the treatment pathway of patients in subgroups B and C.

A flow diagram indicating the treatment pathways of patients in subgroups B and C is shown in Figure 2. One patient in group B and two patients in group C achieved a complete response by liver MRI. Liver resection was deferred in these patients. Radiofrequency ablation (RFA) was used in a total of four patients. Two patients in subgroup B received RFA: one in conjunction with surgery and one as an alternative to surgery (liver resection was abandoned as a result of peri-operative bleeding that occurred while resecting the primary during a combined procedure). In subgroup C, one patient declined surgical resection and opted for RFA as an alternative. A further patient with significant co-morbidity received RFA as the risk of surgery was felt to be high. A two-stage resection with portal vein embolisation was used in two patients. One patient in subgroup C had both stages performed successfully. In subgroup B, the patient developed disease progression shortly after the first stage of the planned two-stage resection. Macroscopic disease clearance was not achieved in this case. Histopathological examination of resection specimens revealed clear margins in 96% (eight out of nine in subgroup B and all 19 in subgroup C) of cases. Pathological complete response was found in two cases, one in each of subgroups B and C.

In subgroup C, a total of 23 patients (72%) achieved either liver resection, RFA (with curative intent) or a complete radiological response by MRI. In subgroup B, the corresponding figure is 11 patients (50%). There were no post-operative deaths related to liver surgery.

### Survival

**Figure 3:**
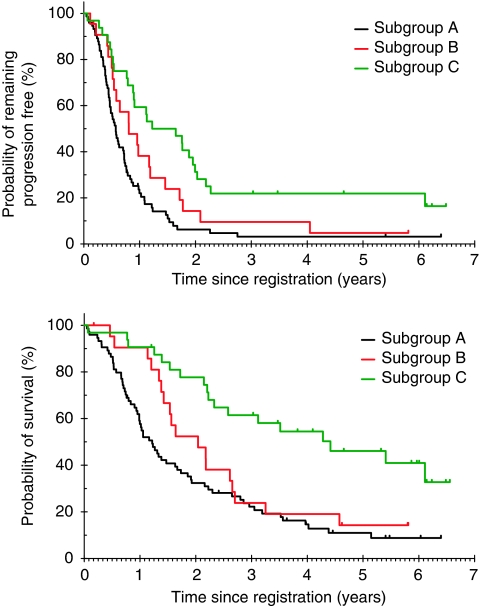
Progression-free survival and overall survival by subgroup.

At a median follow-up of 60 months, 100 (78.1%) patients have died. One patient in subgroup A was not evaluable for PFS as they received second-line irinotecan without documentation of progressive disease. The median overall survival for patients in subgroups A, B and C was 14.6, 24.5 and 52.9 months, respectively. Overall survival and progression-free survival outcomes for the total patient population and for each of the subgroups are shown in Table 3. Supplementary Figure 3 shows the Kaplan–Meier plot for overall survival for all enrolled patients. Overall survival and progression-free survival curves for each patient subgroup are shown in Figure 3.

Of the 29 patients in subgroups B and C who underwent an attempt at curative liver resection, six (20.7%) remain disease free, all from subgroup C. Supplementary Figure 5 shows the progression-free survival and overall survival for subgroup B and C patients who attempted curative resection. The median PFS for these patients with that attempt resection was 14.0 months in subgroup B and 24.3 months in subgroup C. The corresponding median overall survival was 31.3 months in subgroup B and 73.3 months in subgroup C.

## Discussion

Stage IV colorectal cancer encompasses a heterogeneous patient population in which both palliative and curative treatment strategies may be used. In this prospective study, we stratified patients with stage IV disease into three subgroups according to the feasibility of undertaking curative liver resection. All enrolled patients received treatment according to a protocol-defined strategy, with the planned delivery of eight cycles of CapOx chemotherapy and liver resection considered after four or eight cycles of treatment, where feasible. The comparative outcomes of each subgroup were in keeping with our previous expectations. Subgroup C had the highest proportion of patients attempting liver resection, with 59% compared with 45 and 4% in subgroups B and A, respectively. As would be expected, overall survival was also the longest in subgroup C, with a median of 52.9 months compared with 24.5 and 14.6 months for patients in subgroups B and A, respectively. Examining the outcomes of only those patients who underwent liver resection, we found that patients in subgroup C continued to fare better than those in subgroup B. Seven (70%) patients in subgroup B developed progressive or recurrent disease within 12 months of surgery compared with only five (28.5%) in subgroup C.

The stratification method adopted in this study was not based on strictly defined criteria. Although this is a potential criticism, we believe that the stratification method used is representative of clinical practice in which factors such as the status of the primary tumour may affect patient management. What is clearly demonstrated by the results of this study is that even within the subgroup of stage IV patients with liver-only metastases (subgroups B and C), marked variation in patient outcome can be seen. The difference in patient outcomes between subgroups B and C can be understood in light of the differing disease characteristics between subgroups (Table 1). Patients in subgroup B had more numerous liver metastases and were also noted to have a shorter median interval from primary diagnosis to the development of metastases. The disparity in disease characteristics between subgroups remained in those patients who underwent an attempt at curative resection (Supplementary Table 5). These characteristics are known adverse risk factors for disease recurrence following hepatic resection (Schlag *et al*, 1990; Sato *et al*, 1998; Fong *et al*, 1999; Iwatsuki *et al*, 1999; Tsai *et al*, 2007; Rees *et al*, 2008) and are likely to account for the inferior outcomes seen in subgroup B. Data reported by Adam *et al* (2004a) has similarly shown inferior survival outcomes for patients undergoing resection after conversion therapy, compared with those with disease amenable to primary liver resection.

To further examine for potential baseline prognostic factors, an exploratory univariate and multivariate Cox regression analysis was undertaken (Supplementary Tables 6 and 7). For the entire patient cohort, stratification to subgroup C, alk phos <300 and absence of peritoneal disease were found to be independently predictive of both PFS and OS. In the liver-only patient subgroup (B and C), no independently significant prognostic factors for PFS were identified. Stratification to subgroup B and age >60 years were noted to be independently predictive of shorter overall survival.

In view of the wide variation in survival outcomes seen among patients presenting with stage IV colorectal cancer, modification of the AJCC staging system (Greene *et al*, 2002) to allow the sub-categorization of stage IV patients has been proposed (Nagashima *et al*, 2006; Poston *et al*, 2006, 2008; Nordlinger *et al*, 2007). Currently, there remains no widely accepted method of sub-classification; however, our data would lend support to the suggested incorporation of liver resectability status in a revised colorectal cancer-staging system (Nordlinger *et al*, 2007).

A strength of our study data is that the resectability status of each patient was identified prospectively at study entry, thus avoiding the potential pitfalls associated with retrospective classification. The proportion of enrolled patients with liver only disease was higher than would be expected at 42%. It is likely that funding restrictions that applied to the use of oxaliplatin during the period of the study (NICE, 2002) would have biased enrolment towards patients with liver only disease and may have also resulted in the underrepresentation of patients with operable metastases at other visceral sites. This factor should not have influenced the characteristics or the comparative outcomes of the individual patient subgroups.

At the time of initiating this study, there were no data to support the use of neoadjuvant chemotherapy in patients with resectable liver metastases. The subsequently published results of the EORTC 40983 study now lend support to this approach (Nordlinger *et al*, 2008). Although the 40983 study did not show a significant progression-free survival benefit with peri-operative chemotherapy on an intention to treat basis, an improvement in progression-free survival of 9.2% at 3 years was seen in those patients who achieved surgical resection. An additional noteworthy finding in this study was an increase in the post-operative complication rate in patients who received peri-operative chemotherapy at 25 *vs* 16% in the surgery-alone arm (Nordlinger *et al*, 2008). Data suggest that the choice of chemotherapeutic agents (Vauthey *et al*, 2006), length of pre-operative therapy (Aloia *et al*, 2006; Karoui *et al*, 2006) and interval between chemotherapy and surgery (Welsh *et al*, 2007) may all influence the associated surgical morbidity. The relatively short interval between chemotherapy and surgery in the EORTC study (median 4.1 weeks) may have contributed to the excess surgical complication rate seen.

Together with enabling cytoreduction, a further advantage of pre-operative chemotherapy is in allowing for an assessment of chemosensitivity, a marker of underlying disease biology (Charnsangavej *et al*, 2006). Progression on chemotherapy is an indicator of poor outcome following hepatic resection (Adam *et al*, 2004b) and may be used to aid the selection of appropriate surgical candidates. In our study, the length of pre-operative chemotherapy used in subgroup B was relatively short, with a median number of four pre-operative treatment cycles. This is a shorter period of pre-operative treatment than that used in other studies evaluating conversion therapy (Bismuth *et al*, 1996; Alberts *et al*, 2005; Masi *et al*, 2006) in which the duration of chemotherapy was typically 6 months. Our treatment policy of early resection may have contributed to the relatively high rate of early post-operative failure seen in subgroup B. In this high-risk group, a more prolonged period of chemotherapy may have aided the selection of a better prognosis patient group for resection by the exclusion of those who progress while receiving chemotherapy.

Advances in surgery have significantly influenced this field of practice with the criteria for disease resection becoming increasingly broad (Poston *et al*, 2006; Nordlinger *et al*, 2007; Pawlik *et al*, 2008). Consequently it is now possible to undertake liver resection in a greater proportion of patients with adverse disease features who are at higher risk of early post-operative failure. A variety of clinical prognostic scoring systems have been proposed (Fong *et al*, 1999; Iwatsuki *et al*, 1999; Adam *et al*, 2004a; Malik *et al*, 2007; Arru *et al*, 2008; Kattan *et al*, 2008) to aid the identification of patients at higher risk for disease recurrence and allow treatment to be tailored accordingly. It is envisaged that advances in molecular medicine will further identify reliable markers of disease biology, thus enhancing our ability to predict both patient outcome and individual treatment response (Charnsangavej *et al*, 2006; Neal *et al*, 2006; Pawlik and Choti, 2007; Amado *et al*, 2008). The availability of robust methods to assess disease biology will further enable a personalised approach to therapy, allowing the rational application of both surgery and chemotherapy in an individual patient and minimising the exposure to morbid inventions.

## Conclusion

The results of this prospective study illustrate the wide variation in patient outcome according to baseline liver resectability status and highlight the potential value a revised staging system may have in clinical practice.

## Figures and Tables

**Table 1:** Patient characteristics

	**All patients**	**Subgroup A**	**Subgroup B**	**Subgroup C**
Patient number	128	74	22	32
Median age (range)	62 (29–78)	61 (29–78)	68 (38–77)	59 (47–76)
Male (%)	77 (60)	44 (59)	12 (52)	21 (66)
PS 2 (%)	10 (8)	9 (12)	1 (4)	0 (0)
Primary *in-situ* at study entry		55%	41%	28%
Metachronous presentation of >12 months		27%	9%	44%
aSynchronous presentation			86%	53%
Median number of liver deposits (range)			4 (1–15)	2 (1–5)

a Synchronous presentation defined as the development of metastatic disease within 3 months of primary diagnosis.

**Table 2:** Patient outcomes

	**All patients *n*=128**	**Subgroup A *n*=74**	**Subgroup B *n*=22**	**Subgroup C *n*=32**
Median number of cycles (range)	8 (1–12)	8 (1–8)	8 (1–12)	8 (1–8)
CT response rate CR/PR (%)	52%	47%	59%	59%
Attempt at curative resection (%)	32 (25)	3 (4)	10 (45)	19 (59)

**Table 3:** Survival outcomes

	**All patients**	**Subgroup A**	**Subgroup B**	**Subgroup C**
Patient number	128	74	22	32
Median PFS months	8.7	6.9	9.7	14.7
Median OS months	20.7	14.6	24.5	52.9
3-year OS (95% CI)	32.4% (24–41)	22.2% (13–32)	23.8% (9–43)	61.5% (42–76)
