# Supplementary material for: Defining patient outcomes in stage IV colorectal cancer: a prospective study with baseline stratification according to disease resectability status
Source: Br J Cancer. 2010 Jan 19;102(2):255–61. doi: 10.1038/sj.bjc.6605508 (PMC2816665; doi:10.1038/sj.bjc.6605508)
Supplement: Supplementary Information [file 6605508x1.doc]

**Online only - Table 2: Chemotherapy related toxicities**

|  | **All grades %** | **Grade III/IV %** |
| --- | --- | --- |
| **Diarrhoea** | 64 | 14 |
| **Peripheral neuropathy** | 96 | 2 |
| **Hand foot syndrome** | 48 | 6 |
| **Neutropenia** | 55 | 8 |
| **Thrombocytopenia** | 26 | 2 |
| **Febrile neutropenia** | 0 |  |

**Online only - Figure 3: Kaplan-Meier plot of overall survival for all enrolled patients**

**Online only - Figure 5: Progression-free survival and overall survival for patients undergoing attempt at curative resection**

**Online only - Table 5: Characteristics of patients undergoing attempt at resection**

|  | **Subgroup B**  **n=10 (%)** | **Subgroup C**  **n=19 (%)** |
| --- | --- | --- |
| **Median age**  **(range)** | 68  (38-75) | 59  (51-70) |
| **Synchronous**  **Presentation** | 80% | 32% |
| **Primary in-situ** | 4 (40) | 4 (21) |
| **Received long-course pelvic chemoradiotherapy** | 2 (20) | 1 (5) |
| **Median time from diagnosis of primary site to presentation with metastases** | 1.8 months | 16.1 months |
| **Metachronous presentation**  **>12 months** | 10% | 68% |
| **Median number of liver lesions at baseline for resected patients** | 3.5  (range 1-7) | 2  (range 1-5) |
| **Mean diameter of largest resected metastasis** | 27.1mm  (range 0-65) | 20.5mm  (range 0-55) |
| **Type of liver resection** |  |  |
| **Right Hemihepatectomy** | 0 | 3 |
| **Extended R Hepatectomy** | 2 | 2 |
| **Left hemihepatectomy** | 2 | 2 |
| **Extended L hepatecomy** | 2 | 3 |
| **Segmentectomy**  **– 1 segment** | 2 | 3 |
| **Segmentectomy**  **– 2 or 3 segments** | 1 | 6 |
| **RFA** | 1 | 0 |

Table 6. Univariate and multivariate predictors of progression-free survival for all enrolled patients.

|  | **Univariate Analysis** | | | | **Multivariate Analysis** | | | |
| --- | --- | --- | --- | --- | --- | --- | --- | --- |
|  | **Sig.** | **HR** | **95.0% CI for HR** | | **Sig.** | **HR** | **95.0% CI for HR** | |
|  |  |  | **Lower** | **Upper** |  |  | **Lower** | **Upper** |
| **Primary disease** | **.007** | **1.68** | **1.15** | **2.43** | NS | - | - | - |
| **Local disease** | **.017** | **1.98** | **1.13** | **3.47** | NS | - | - | - |
| **No. metastases > 1** | **.003** | **1.82** | **1.23** | **2.70** | NS | - | - | - |
| **DFI > 12 months** | **.010** | **0.57** | **0.37** | **0.88** | NS | - | - | - |
| **Synch presentation** | **.020** | **1.61** | **1.08** | **2.42** | NS | - | - | - |
| **PS = 2** | **.012** | **2.37** | **1.21** | **4.63** | NS | - | - | - |
| **Hb <11** | **.018** | **1.78** | **1.11** | **2.87** | NS | - | - | - |
| **WCC >10** | **.022** | **1.77** | **1.09** | **2.88** | NS | - | - | - |
| Subgroup B | .087 | 0.65 | 0.39 | 1.07 | NS | - | - | - |
| **Subgroup C** | **.000** | **0.38** | **0.24** | **0.60** | **.001** | **.460** | **.290** | **.740** |
| **Alk phos >300** | **.000** | **3.51** | **1.78** | **6.93** | **.002** | **2.98** | **1.50** | **5.94** |
| **Peritoneal disease** | **.015** | **2.16** | **1.16** | **4.01** | **0.47** | **1.89** | **1.01** | **3.55** |

**Univariate and multivariate predictors of overall survival for all enrolled patients.**

|  | **Univariate Analysis** | | | | **Multivariate Analysis** | | | |
| --- | --- | --- | --- | --- | --- | --- | --- | --- |
|  | **Sig.** | **HR** | **95.0% CI for HR** | | **Sig.** | **HR** | **95.0% CI for HR** | |
|  |  |  | **Lower** | **Upper** |  |  | **Upper** | **Upper** |
| **Primary disease** | **.023** | **1.59** | **1.07** | **2.36** | NS | - | - | - |
| **Local disease** | **.008** | **2.22** | **1.23** | **4.00** | NS | - | - | - |
| **No. metastases > 1** | **.014** | **1.68** | **1.11** | **2.54** | NS | - | - | - |
| **PS = 2** | **.001** | **3.07** | **1.56** | **6.02** | NS | - | - | - |
| **Hb <11** | **.025** | **1.78** | **1.08** | **2.96** | NS | - | - | - |
| **Platelets < 400** | **.043** | **0.61** | **0.38** | **0.99** | NS | - | - | - |
| **Bone disease** | **.032** | **4.76** | **1.14** | **19.87** | **.024** | **5.37** | **1.25** | **23.11** |
| **WCC >10** | **.001** | **2.35** | **1.42** | **3.90** | **.022** | **1.92** | **1.10** | **3.37** |
| Subgroup B | .189 | 0.70 | 0.42 | 1.19 | NS | - | - | - |
| **Subgroup C** | **.000** | **0.32** | **0.19** | **0.55** | **.001** | **.409** | **.239** | **.699** |
| **Alk Phos >300** | **.000** | **6.95** | **3.33** | **14.51** | **.000** | **4.50** | **2.03** | **9.96** |
| **Peritoneal disease** | **.003** | **2.54** | **1.36** | **4.71** | **.013** | **2.24** | **1.18** | **4.24** |

Table 7. Univariate factors predictive of progression-free survival in patients with isolated liver metastases (subgroups B &C).

|  | **Univariate Analysis** | | | |
| --- | --- | --- | --- | --- |
|  | **Sig.** | **HR** | **95.0% CI for HR** | |
|  |  |  | **Lower** | **Upper** |
| Largest liver met > 5cm | .425 | 1.35 | .65 | 2.80 |
| No. liver mets >1 | .471 | .80 | .43 | 1.48 |
| CEA >200 | .480 | .71 | .28 | 1.82 |
| WCC >10 | .809 | 1.12 | .44 | 2.86 |
| Hb <11 | .099 | 1.93 | .89 | 4.20 |
| Platelets <400 | .765 | 1.12 | .52 | 2.41 |
| LDH >ULN | .921 | 1.05 | .41 | 2.71 |
| **DFI >12 months** | **.020** | **0.46** | **0.23** | **0.88** |
| **Synch presentation** | **.046** | **1.90** | **1.01** | **3.58** |
| Subgroup B | .063 | 1.76 | .97 | 3.20 |
| Age >60 | .066 | 1.76 | .96 | 3.22 |

Univariate and multivariate predictors of overall survival in patients with isolated liver metastases (subgroups B &C).

|  | **Univariate Analysis** | | | | **Multivariate Analysis** | | | |
| --- | --- | --- | --- | --- | --- | --- | --- | --- |
|  | **Sig.** | **HR** | **95.0% CI for HR** | | **Sig.** | **HR** | **95.0% CI for HR** | |
|  |  |  | **Lower** | **Upper** |  |  | **Lower** | **Upper** |
| Largest liver met > 5cm | .954 | 1.03 | .425 | 2.48 | NS | - | - | - |
| No. liver mets >1 | .987 | .994 | .493 | 2.00 | NS | - | - | - |
| CEA >200 | .854 | .906 | .317 | 2.59 | NS | - | - | - |
| WCC >10 | .809 | 1.14 | .400 | 3.23 | NS | - | - | - |
| Hb <11 | .730 | 1.18 | .458 | 3.05 | NS | - | - | - |
| Platelets < 400 | .112 | 2.35 | .818 | 6.77 | NS | - | - | - |
| LDH >ULN | .541 | 1.45 | .438 | 4.83 | NS | - | - | - |
| DFI > 12 months | .432 | .751 | .368 | 1.53 | NS | - | - | - |
| Synch presentation | .771 | 1.11 | .559 | 2.19 | NS | - | - | - |
| **Subgroup B** | **.012** | **2.37** | **1.21** | **4.63** | **.011** | **2.45** | **1.23** | **4.88** |
| **Age >60** | **.022** | **2.31** | **1.13** | **4.72** | **.013** | **2.54** | **1.22** | **5.33** |
